# Male‐Biased Adult Mortality in the Great Bustard Is Consistent With High Reproductive Costs and Aggravated by Anthropogenic Impact

**DOI:** 10.1002/ece3.72713

**Published:** 2026-01-07

**Authors:** Juan C. Alonso, Beatriz Martín, Carlos Palacín, Carlos A. Martín, Javier A. Alonso

**Affiliations:** ^1^ Department of Evolutionary Ecology Museo Nacional de Ciencias Naturales (MNCN), CSIC Madrid Spain; ^2^ Randbee Consultants C/Carretería 67 Málaga Spain; ^3^ Department of Biodiversity, Ecology and Evolution, Faculty of Biological Sciences Universidad Complutense Madrid Spain

**Keywords:** adult sex ratio, annual survival, great bustard, human‐induced mortality, *Otis tarda*, sexual selection

## Abstract

Sex differences in adult mortality have usually been explained as a result of differences between males and females in the costs associated with their reproductive investment. Investigating sex‐biased mortality is important because it shapes mating opportunities, reproductive strategies and parental care. Here, using known fate models implemented in the RMark package, we estimate annual and monthly or seasonal adult survival rates in a sample of 339 great bustards ( *Otis tarda* ) radio‐tagged in 1985–2013 and monitored up to 2020. We found that annual survival was lower in males than in females, and lower in Madrid, a highly anthropized region (males: 0.874, females: 0.931), than in Villafáfila, where very good habitat conditions still exist (males: 0.948, females: 0.973). The maximum ages reached by marked individuals were also higher in females (17.3 years) than in males (13.8 years). The seasonal survival pattern was similar in both sexes, with maximum mortality at the end of the mating and incubation period, and lower survival in males, suggesting a direct effect of reproductive cost in both sexes and a higher cost in males. These results are consistent with previous comparative studies, which attribute male‐biased adult mortality to the cost of mating competition in males and egg productivity in females, although these hypotheses could not be tested in the present study. Anthropogenic mortality was considerable (39.5% of deaths) and also male‐biased, with power line casualties standing out and affecting 3.5% of males and 1.9% of females each year in Madrid. Anthropogenic mortality thus contributes to aggravate the naturally biased survival rates and could be a major cause of the declines observed in most great bustard populations over the last decades.

## Graphical abstract

Annual survival of great bustards ( *Otis tarda* ) was lower in males than in females, with minimum values just after the mating and incubation period, respectively, a pattern that is consistent with the reproductive cost hypothesis. Survival was lower in Madrid, a highly anthropized region (males: 0.874, females: 0.931), than in Villafáfila, where good habitat conditions still exist (males: 0.948, females: 0.973). Anthropogenic mortality was considerable (39.5% of deaths), and also male‐biased, with power line casualties standing out, and affecting 3.5% of males and 1.9% of females each year in Madrid. Anthropogenic mortality thus contributes to aggravate the naturally biased survival rates and could be a major cause of the declines observed in most great bustard populations over the last decades.

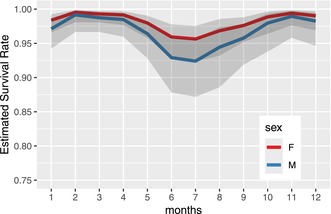

## 1 Introduction

Sex differences in adult mortality have generally been attributed to the costs associated with the reproductive roles of each sex (Liker and Székely 2005; Moore and Wilson 2002; Owens and Bennett 1994; Promislow 1992; Roff 2002). For example, while in most birds mortality is female‐biased due to the heavy investment of females in egg production (*egg productivity hypothesis*; Romano et al. 2022) or in nesting and rearing young (*parental care hypothesis*; Donald 2007; Monaghan et al. 1998; Owens and Bennett 1994; Promislow et al. 1992; Sibly et al. 2012), the male‐biased adult mortalities in most mammals are attributed to intense male–male competition (*sexual selection hypothesis*; Clutton‐Brock 1991; Promislow 1992). These studies provide a general idea of mortality patterns through correlational evidence, but they do not clarify much about the causes and mechanisms that produce sex bias in mortality. One such cause may be the difference between sexes in the prevalence of parasites or immune response capacity (Klein and Flanagan 2016; Moore and Wilson 2002; Valdebenito et al. 2020; Zuk 2009). Within the framework of sexual selection and life history theory, it has been proposed that the lower immune response capacity of males in many species is a consequence of their greater investment in mating success (Kelly et al. 2018; Moore and Wilson 2002; Valdebenito et al. 2021; Vincze et al. 2022; Zuk 2009). However, the results are unclear in birds. For example, Valdebenito et al. (2020) found no evidence that parasite prevalence is a good predictor of sex differences in mortality in birds, and other studies even question the existence of sex‐different immune response in birds (Foo et al. 2017; Roberts et al. 2004). Valdebenito et al. (2020) suggest that factors such as exposure to predators, immune capacity and cost of reproduction need to be analysed together, and call for further comparative and single‐species studies to understand the causes of sex‐specific mortality in wild bird populations.

Sex biases in adult mortality skew the mating pool (Székely, Liker, et al. 2014), influencing mate competition, parental strategies, and ultimately key demographic processes in a population (Kokko and Jennions 2008; McNamara et al. 2000; Székely, Weissling, and Komdeur 2014). Therefore, it is important to have a good understanding of the relationships between adult sex ratio, adult mortality and reproductive investment. A better comprehension of the mechanisms that determine sex‐biased mortality and its immediate consequence, the skewed adult sex ratio, helps to explain why males and females have different survival strategies and lifespans, and how these differences can shape mating systems and social structure, as well as ultimately the dynamics and demographics of the population (Alonzo and Sheldon 2010; Székely et al. 2000; Székely, Weissling, and Komdeur 2014).

In this study, we provide the first detailed quantification of sex‐specific adult survival in the great bustard ( *Otis tarda* ), and we interpret our findings in light of existing hypotheses about reproductive costs and sex‐biased mortality. Our study does not aim to test the hypotheses of sexual selection, egg productivity and parental care, but rather use them as an interpretative framework. Great bustards show the most extreme documented sexual size dimorphism in wild flying birds, with males more than twice the size of females (Alonso, Magaña, et al. 2009), a trait likely shaped by intense sexual selection through male–male competition and female choice (Alonso et al. 2010; Magaña, Alonso, Alonso, et al. 2011). At the same time, females bear all parental responsibilities, from incubation to chick rearing, often for over a year (Alonso et al. 1998, 2018). On the other hand, given that mortality in birds during migratory journeys has been found to be much higher than during stationary periods (Newton 2025), we considered as an alternative hypothesis that migration costs could be more important than reproductive costs in determining sex differences in mortality. In Iberia, males migrate mainly in summer and females in winter (Palacín et al. 2009). Therefore, if migration were the main driver, mortality should peak in each sex during their respective migratory periods.

Our study is the first to provide data on adult survival of the great bustard, based on a large sample of marked individuals. Our objectives were: (1) To quantify adult mortality in both sexes and evaluate whether observed sex differences in survival are more consistent with costs of sexual selection acting on males than with costs of egg productivity and parental care acting on females (Romano et al. 2022; Liker and Székely 2005). (2) To examine whether the seasonal pattern of mortality in each sex better reflects the cost of reproductive investment or that of migration (summer migration in males, winter migration in females). (3) To determine and quantify the causes of mortality in this species, both natural and anthropogenic, in order to evaluate whether sex‐biased mortality reflects reproductive costs or sex‐biased exposure to human‐induced threats. We gave special attention to identifying anthropogenic mortality causes, which are currently responsible for a high percentage of deaths in many vertebrates (Hill et al. 2019; Loss et al. 2015). We assessed the importance of anthropogenic mortality by comparing it with natural mortality, and by comparing natural and anthropogenic causes in two populations subjected to different degrees of human influence (Madrid, a region with dense human infrastructure and high anthropogenic pressure, and Villafáfila, a region with much better quality habitat and less human‐induced disturbance). Contrasting Madrid and Villafáfila provides a natural test of context‐dependent sex‐biased mortality, because the two regions differ sharply in extrinsic risks while holding species biology constant. Theory predicts that the sex investing more heavily in costly reproductive tactics—such as males engaging in energetically expensive displays or risky movements to access mates—should experience disproportionately higher mortality when background hazards rise (sensu Kokko and Jennions 2008). Thus, if anthropogenic pressures in Madrid amplify exposure to human‐induced mortality causes, disturbance, or energetically demanding avoidance behaviour, they should magnify the pre‐existing vulnerability of the sex with higher reproductive costs, steepening sex differences in mortality relative to Villafáfila. Conversely, in the lower‐risk environment of Villafáfila, sex‐biased mortality should be weaker, reflecting a closer match between intrinsic reproductive costs and realised survival. This regional contrast therefore allows inference about how environmental harshness interacts with sex‐specific strategies to shape demographic patterns.

Finally, the quantification of anthropogenic mortality causes is particularly important from a biodiversity conservation perspective, as the conservation status of the great bustard has recently been uplisted from *vulnerable* to *endangered* (BirdLife International 2024), due to a global population decline of > 30% in the last two decades (Alonso and Palacín 2022). In Spain, the species is classified as *near threatened* (Palacín and Alonso 2021), with current estimates of 22,000–24,000 individuals, and the population is experiencing ongoing declines (Alonso and Palacín 2022). Both the survival estimates, due to their potential application in demographic studies, and the determination and quantification of mortality rates by different causes, which may guide management measures to help reverse the population decline, are important for the conservation of this endangered species.

## 2 Methods

### 2.1 Study Species

Great bustard males are among the heaviest flying birds in the world, weighing 2.48 times more than females, and showing 18%–30% larger linear measurements (Alonso, Magaña, et al. 2009). This species is originally native to Eurasian steppes, but has adapted to agricultural environments, especially those dominated by dry cereal crops. Its reproductive system is the dispersed lek, a type of polygyny based on the degree of dominance of the males, and there is a female‐biased sex ratio (Alonso and Alonso 1990), in which the two sexes live in separate groups throughout the year (Alonso, Salgado, and Palacín 2016), showing differences in their diet (Bravo et al. 2016). Males gather in late winter at traditional display arenas, where they establish a hierarchy and display to attract females, and only ca. 10% of them copulate each year (Alonso et al. 2010). Year after year they use the same display grounds. After copulations in April, most males leave the lek area between May and July (Palacín et al. 2009, 2017), and females take on all breeding duties (Magaña 2007; Magaña, Alonso, and Palacín 2011). Chicks depend on their mothers throughout their first 6–18 months of life (Alonso et al. 1998). Productivity is low and variable between years and regions and apparently related to winter rainfall, which determines the abundance of food for the chicks (Morales et al. 2002). A female bustard only manages to raise an average of one or two chicks in her lifetime (Magaña 2007; Morales et al. 2002). Juvenile dispersal is male‐biased (Alonso et al. 1998; Martín et al. 2008). In Spain, the species is a partial and sex‐differential migrant, with females from some populations migrating generally southwards up to 120 km in winter, and males mostly in summer, to cooler, rainier areas located up to 250 km north of their breeding sites. Some males return in autumn to their leks, while others migrate to the same wintering areas as females (Alonso, Palacín, et al. 2009; Palacín et al. 2009, 2017). Annual juvenile mortality is high (0.70 in the first year, 0.10 in the second) and male‐biased due to the males' faster growth and increased vulnerability to food shortages (Martín et al. 2007). This results in a female‐biased sex ratio among first‐year birds (Martín et al. 2007), which continues into adulthood, with values ranging from 1.4 to 3.9 females per male (Alonso and Alonso 1990; Alonso, Palacín, et al. 2005, 2016).

### 2.2 Marking and Tracking Birds

We analysed adult survival data from 339 great bustards marked between 1985 and 2013, and monitored over their lifetime until the death of the last individual in 2020 (Table S1). This sample includes only data from the adult age, that is from birds captured as adults or juveniles older than 2.5 and 3.5 years for females and males respectively, when they can be considered adults (Magaña 2007; Martín et al. 2008). We captured young birds between mid July and early August, when they were 4–11 weeks old and weighed on average 1.4 kg (females) and 2 kg (males). We marked the birds on both wings with PVC patagial marks (Gravoply, www.gravograph.es) with an engraved symbol, letter or number that allowed individual identification with a telescope. Although the use of patagial tags could have detrimental effects, we did not observe any negative effects from this tagging method (Martín et al. 2007). The tags were 70 × 65 mm large and 1.5 mm thick. They were attached to the wing patagium using Allflex rivets and ear tag pliers. The total weight of tag plus rivet was ca. 12 g. We covered tags with thin brown paper painted with black imitating the plumage design of the birds to reduce as much as possible the visibility of the tag to predators. The paper usually fell off after some days.

Birds were also equipped with radio transmitters. The main transmitter model was Biotrack TW3 2 × AA backpack‐mounted unit, weighing 60 g, which we used for most young birds, all females and some males. In the first years, a small number of birds were marked with other models or with transmitters from other manufacturers (Telonics CHP‐4P, Telonics 225, 30 g Biotrack TW3 2 × 2/3AA ‘poncho’‐mounted or neck‐lace units, Biotrack TW3 1 × C, and solar‐powered 50 g PTT 100 s satellite units from Microwawe Telemetry Inc., Columbia). The TW3 2 × AA backpack‐mounted units, which were by far the most commonly used, were attached to the bird with an elastic harness to allow for expansion as the young birds grew. The total weight of the transmitter plus harness did not exceed the recommended limit of 3%–5% of the bird's weight (Kenward 2001). In all cases, chicks joined their mother after release following a marking process lasting 10–15 min, with no adverse effects in their immediate behaviour after release. We also found no detrimental effects of marking on the survival of chicks (Martín et al. 2007).

Adults were captured using rocket nets, which was selected as the most appropriate procedure after testing various methods. All adults were fitted with radio transmitters using Teflon or elastic band harnesses, as well as Gravoply wingtags like those used for juveniles. In females and a number of males, these plates were attached to both wing patagiums, while in the rest of the males they were glued vertically on top of the backpack‐mounted transmitter. The advantage of this latter system is the shorter time needed for marking. The transmitters used on adults were all from Biotrack Ltd., and weighed no more than 1% of the bird's weight.

All marked birds were located at least once per month throughout their life, using AVM LA 12‐DS telemetry receivers (first few years) or Telonics TR2‐TS1 (rest of years). When the signal was lost from the ground, we used military aircraft from the Spanish Air Forces (E‐24 Bonanza, Beechcraft, Wichita, Kansas) for aerial location, which increased the reception range of the transmitter signals by 10–20 times and allowed us to survey much larger areas. Aerial locations were close to 100% successful, and the average location error from the air did not exceed 500 m. The total flight time during this study was 2544 h. The batteries of the backpack‐mounted transmitters used in most chicks and adult females (TW5 2xAA) lasted between 4 and 5 years, and those used in most adult males (100 g TW5 3 × AA) lasted 6–8 years.

### 2.3 Study Areas

The study was carried out in several regions of Spain, where great bustards were captured in 29 breeding groups or *leks* (Figure S1). These regions are located in the Mediterranean bioclimatic area, between parallels 37^o^0′N–43^o^0′N and meridians 0^o^0′W–8^o^0′W. According to the latest published estimate, the Spanish great bustard population represents 68%–72% of the world total of this species (Alonso and Palacín 2022). The two main study regions, where most individuals were marked, were Madrid and Villafáfila. In other regions, smaller numbers of birds were marked (Albacete, Ávila, Burgos, Córdoba, Navarra, Salamanca, Sevilla, Toledo, Valladolid, Zaragoza) (Table S1). In all these regions, great bustards live in flat or slightly undulating, treeless areas, mainly used for dry cereal cultivation, with alternating scattered fallows, pastures and some other crops (alfalfa, vineyards, olive trees). The climate is characterised by hot, dry summers, rainy springs and autumns and cold winters.

The region named Villafáfila comprises the Special Protection Area for Birds ‘Lagunas de Villafáfila’ (Red Natura 2000, ES0000004) and its surroundings, totalling some 57,000 ha in the north‐east of Zamora province (central coordinates 41^o^50′N, 5^o^36′W). This region has the world's highest great bustard densities, with 4–6 individuals per km^2^, and a population of about 2200–2400 birds distributed in 11 leks (mean 1987–1998 = 1955, maximum = 2174; Morales 1999; mean 1998–2023 = 2412, maximum = 2608; Junta de Castilla y León 2024). This area represents a high‐quality habitat for the great bustard, with a sparse human population, distributed in about 20 small villages, connected by roads with little traffic.

The region called Madrid contrasts with the previous one due to its high human influence. Located between 20 and 60 km from the city of Madrid, it is home to a large human population, busy roads, industrial areas, residential areas and other infrastructure. Despite this development, it comprises four Special Protection Areas for Birds (‘Estepas Cerealistas de las Cuencas de los ríos Jarama y Henares’, ES0000139, ‘Cortados y Cantiles de los ríos Jarama y Manzanares’, ES0000142 and ‘Carrizales y Sotos de Aranjuez’, ES0000119, in Madrid province; and ‘Estepas Cerealistas de La Campiña’, ES0000167, in Guadalajara province). The great bustard population in Madrid was around 1600 individuals a few years ago, distributed in 21 leks, although in the last two decades it has experienced a ca. 40% decline (Alonso et al. 2003; Palacín and Alonso 2022).

### 2.4 Cause of Death Determination

To determine the cause of death, a necropsy was performed when possible. However, in most cases, the degree of decomposition of the carcass due to the high temperatures in our study areas did not allow a precise determination of the cause of death. In those cases, we used criteria described in similar studies, such as the presence of predator tracks near the carcass, and damage from their bites on feathers and bones, or presence of the carcass under or near power lines or roads, with clear evidence of collision (Martín et al. 2007; Palacín et al. 2017; Wolfe et al. 2007). We classified the cause of death into the following classes:

#### 2.4.1 Natural Causes

(a) Natural death without predation: The body was found complete, without signs of predation, with the transmitter and wingtags in place. Most individuals included in this category probably died from starvation, disease, infection, or senescence. In this category we also included all deaths in which any anthropogenic cause was discarded. Therefore, this category included causes that were unknown, but for which we could be reasonably sure that they were due to natural processes (excluding predation), that is not anthropogenic.

(b) Predation: The carcass was partially eaten, usually with head and chest missing. We distinguished between dog and fox as predators on the basis of the traces left (tracks, scent). Although some of these birds could have been scavenged postmortem, we believe that most cases classified in this category were truly predated.

#### 2.4.2 Human‐Induced Causes

(c) Hunting: We found pellets through radiography, or the holes of the shot were clearly visible in the body or wingtag. In some cases the head and neck were missing, like in the past when hunting was legal, when they were cut off by hunters to prepare them as trophies, similar to the way big game trophies are prepared. In a few cases we found only the harness cut with a knife, most times without any remains of the bird. In these cases, neither transmitter nor harness had a putrid smell, which indicated that they had been separated from the bird before rotting started, that is just after having been shot.

(d) Collision with power line/telephone line/wire fence: The carcass was found below an electric or telephone line, or beside a wire fence, usually with broken bones or neck or breast showing cuts or signs of feather abrasion caused by the cables/wires.

(e) Roadkill or collision with train: The carcass was found on a road or railway track, with obvious signs of having collided with a car or train.

The date of death was estimated based on the degree of decomposition of the carcass and on our experience of cases with a known date of death. When only remains were found, such as a few feathers or bones, or only the transmitter, the date of death was estimated as the mean between the last time the bird had been observed alive and the date the remains were found. The interval between both dates did not exceed 30 days.

### 2.5 Statistical Analyses

We explored both the effect of sex as well as the impact of migration in survival using known fate models (White and Burnham 1999). Best‐supported models were compared using the corrected Akaike information criterion for small sample sizes (AICc, Hurvich and Tsai 1989) in order to find the most parsimonious model (the one with the lowest ΔAICc and the smallest number of parameters). As models best supported by empirical data we considered those with AICc differing from the minimal one (i.e., ΔAICc) by < 2 units (Burnham and Anderson 2002). R package RMark (Laake 2014) was used for all the computations.

#### 2.5.1 Effect of Sex on Survival

To assess sex differences in survival, both annual and monthly adult survival rates were estimated.

##### 2.5.1.1 Effect of Sex: Annual Survival

Annual survival estimates were based on data provided by 274 adult birds (females older than 2.5 and males older than 3.5 years) marked in the two main study regions (106 females and 102 males in Madrid, 35 females and 31 males in Villafáfila). These two regions were used as a grouping factor in the models where annual survival rates were estimated.

##### 2.5.1.2 Effect of Sex: Monthly Survival

Monthly survival rates were also estimated on the adult sample only for birds marked and monitored at least over 4 years since marking, to ensure a representative sample of full monthly data over the year for each individual in the sample. In addition, considering 4‐year capture‐recapture data for all the individuals in the sample allowed avoiding that particular months and/or individuals would be overrepresented/underrepresented in the sample. Due to the reduced sample in other regions fulfilling the previous conditions, monthly survival rates were only estimated in the Madrid region based on capture‐recapture records of 106 females and 102 males.

##### 2.5.1.3 Effect of Sex: Seasonal Survival

To obtain comparable estimates between Madrid and Villafáfila over the year, monthly data were grouped into seasons (winter: December–February; spring: March–May; summer: June–August; and autumn: September–November) and seasonal survival estimates were derived for both sexes in both regions (sample size: 106 females and 102 males; 36 females and 30 males, in Madrid and Villafáfila, respectively).

#### 2.5.2 Effect of Migration on Survival

We explored the impact of migration in males (measured through the maximum migration distance performed by each individual between the breeding and summering or wintering areas) on annual survival. Specifically, we used data provided by 195 adult males in all the study regions (Table S1; Figure S1). In these models, we considered latitude as an indirect variable controlling the possible effects of the different breeding areas on survival.

#### 2.5.3 Estimation of Cause‐Specific Mortality Rates

Cause‐specific annual mortality rates were defined as the probability of an individual dying from a given cause of death. To determine specific annual mortality rates for each cause of death, the average annual mortality rate was quantified from survival estimates as (1‐Ŝ_i_) and it was multiplied by the proportion of deaths by each of the previously defined causes (Hebblewhite et al. 2003).

## 3 Results

### 3.1 Annual Survival

The two best models that can be considered plausible (ΔAICc < 2) to describe the annual survival rate of adult great bustards included the effects of sex and region (Table 1). Males showed significantly lower survival than females (β_sexM = −0.565, CI = [−1.018, −0.112]; Table S2). Survival was lower in Madrid than in Villafáfila (β_regionVillafafila = 1.712, CI = [0.276, 3.147]; Table S2), although the interaction between both variables was not significant, supporting an additive effect of sex and region on survival. There was no significant effect of year of marking or year of birth on survival, suggesting that the results were not affected by any other factor intrinsic to year or cohort (Table 1). In both regions, the estimated annual survival of males was significantly lower than that of females (Table 2). Annual survival was lower in Madrid than in Villafáfila in both sexes (Table 2). Regarding the effect of migration distance and breeding site latitude on annual survival of males, models including these variables were not better than the model without them (Table S3). The effect of migration distance on survival was negative but did not reach statistical significance (Figure S2). The maximum ages at which individuals marked as chicks were observed were higher in females than in males: 17 years–3 months–23 days in a female, 13 years–10 months–5 days in a male.

**TABLE 1:** Models for annual survival rates of adult great bustards in Madrid and Villafáfila (*n* = 274 individuals).

	Model	*K*	AICc	ΔAICc	Weight	Deviance
1	S(~sex * region)	4	622.70	0.00	0.389	46.05
2	S(~sex + region)	3	622.91	0.21	0.350	48.27
3	S(~sex)	2	629.11	6.41	0.016	56.48
4	S(~region)	2	630.16	7.46	0.009	57.53
5	S(~sex + markyear)	3	630.68	7.98	0.007	624.66
6	S(~sex * markyear)	4	630.81	8.11	0.007	622.77
7	S(~time)	16	636.28	13.58	0.000	35.17
8	S(~1)	1	637.41	14.71	0.000	66.79
9	S(~markyear)	2	638.87	16.17	0.000	634.86

*Note:* We assessed both interaction and additive models for sex and region (sex * region; sex + region, respectively). The models are ordered from lowest to highest AICc, indicating number of parameters (*K*), AICc, ΔAICc, model weight and deviance.

**TABLE 2:** Estimates of annual survival rates of male and female great bustards in the two regions studied obtained from the most parsimonious best fit model (Table 1, model 2).

	Estimate	SE	Lower CI	UpperCI
Female survival Madrid	0.931	0.011	0.906	0.950
Male survival Madrid	0.874	0.017	0.837	0.903
Female survival Villafáfila	0.973	0.010	0.944	0.987
Male survival Villafáfila	0.948	0.019	0.897	0.974

*Note:* Standard error (SE) and confidence intervals are provided.

### 3.2 Seasonal and Monthly Survival

**FIGURE 1:**
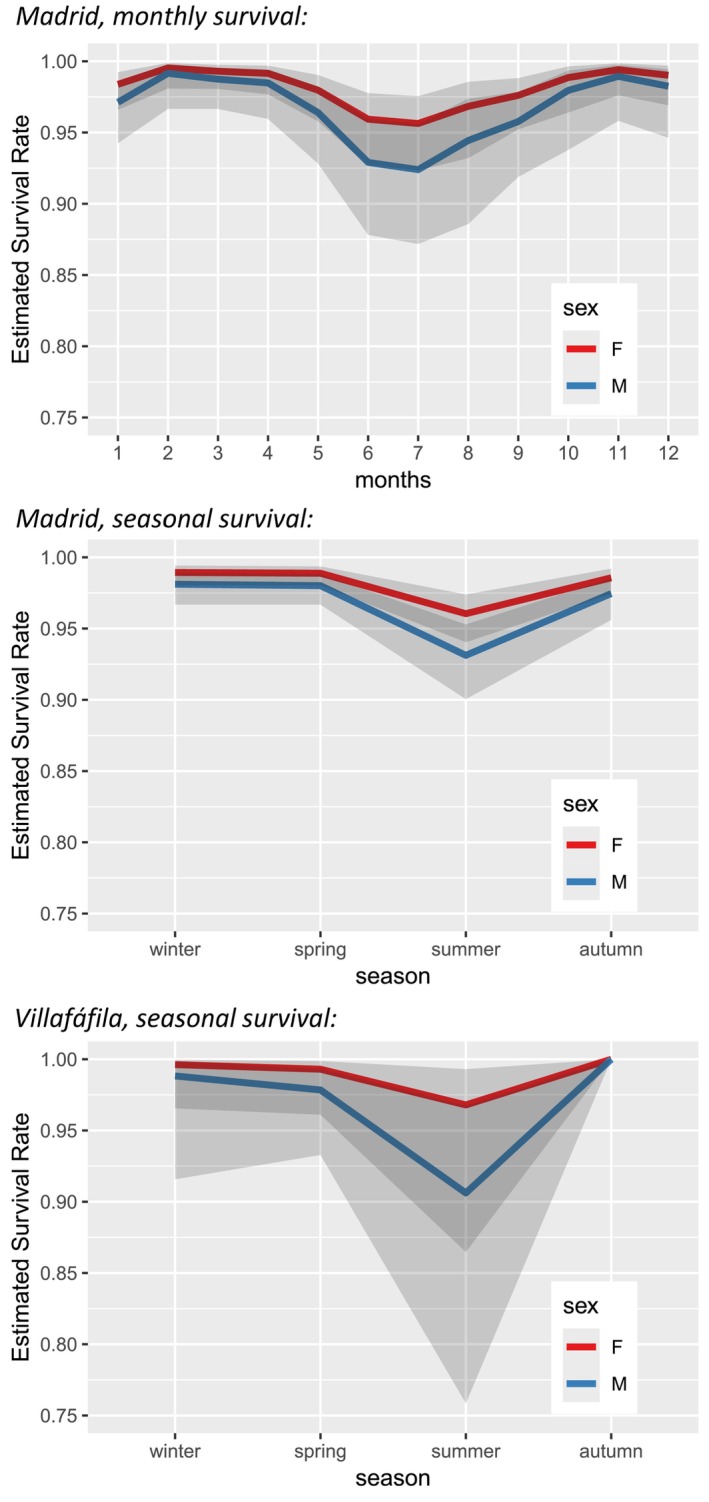
Estimates of mean monthly and seasonal survival in both regions. The sample size for Villafáfila did not allow calculating reliable monthly survival values (results not shown). F, females, M, males. Months from January (month = 1) to December (month = 12). Shadow areas delimit the 95% CIs of the estimates.

The analysis of year‐round variation in mortality provided one plausible model for Madrid and three for Villafáfila (Table 3). These models retained the effect of sex and season in both regions; the models that retained the effect of month exceeded the threshold of ΔAICc < 2 and were thus considered less plausible (model 3 in Madrid, model 6 in Villafáfila; Table 3). The pattern of seasonal variation in survival was similar in both sexes and in the two regions, with maximum values in autumn‐winter and minimum values in early summer (Figure 1). Summer survival was significantly lower than winter survival only in Madrid, with Villafáfila showing the same trend but not reaching statistical significance due to small sample size (Table S4). In the best models including the variable month (Table 3), significant differences were also only detected in Madrid –the region for which a larger sample was available–, between the maximum survival in winter (January) and the minimum in summer (June and July), while no significant differences were found in Villafáfila (results not shown). Male survival was lower than female survival in both regions, although these sex differences were again significant only in Madrid (Table S4).

**TABLE 3:** Models for monthly and seasonal adult survival rates of adult great bustards in the two regions studied.

	Model	*K*	AICc	ΔAICc	Weight	Deviance
**Madrid**						
1	S(~season + sex)	5	710.82	0.00	0.8049	96.18
2	S(~season)	4	715.09	4.27	0.0950	102.46
3	S(~month + sex)	13	715.21	4.39	0.0898	84.47
4	S(~month)	12	719.55	8.73	0.0102	90.83
5	S(~sex)	2	730.77	19.95	0.0000	122.14
6	S(~1)	1	734.13	23.31	0.0000	127.50
7	S(~markyear)	2	736.13	25.31	0.0000	732.12
8	S(~sex + time)	49	749.77	38.95	0.0000	45.64
9	S(~time)	48	754.92	44.10	0.0000	52.86
10	S(~sex * time)	96	802.39	91.57	0.0000	0.00
**Villafáfila**						
1	S(~season + sex)	5	80.98	0.00	0.3822	26.05
2	S(~season)	4	81.08	0.10	0.3642	28.19
3	S(~sex)	2	82.97	1.99	0.1413	34.14
4	S(~1)	1	84.07	3.09	0.0814	37.26
5	S(~markyear)	2	86.09	5.11	0.0297	82.07
6	S(~month)	12	93.62	12.64	0.0007	24.18
7	S(~month + sex)	13	94.04	13.06	0.0006	22.49
8	S(~sex + time)	49	153.67	72.69	0.0000	0.00
9	S(~time)	48	157.39	76.41	0.0000	6.18
10	S(~sex * time)	96	282.90	201.92	0.0000	0.00

*Note:* The models are ordered from lowest to highest AICc, indicating number of parameters (K), AICc, ΔAICc, model weight and deviance. Models test the effects of month and sex on survival rates. We assessed an additive effect for sex and month (*sex + month*). The seasons were defined as follows: Winter = December–February; spring = March–May; summer = June–August; autumn = September–November.

### 3.3 Mortality Rates by Cause of Death

**FIGURE 2:**
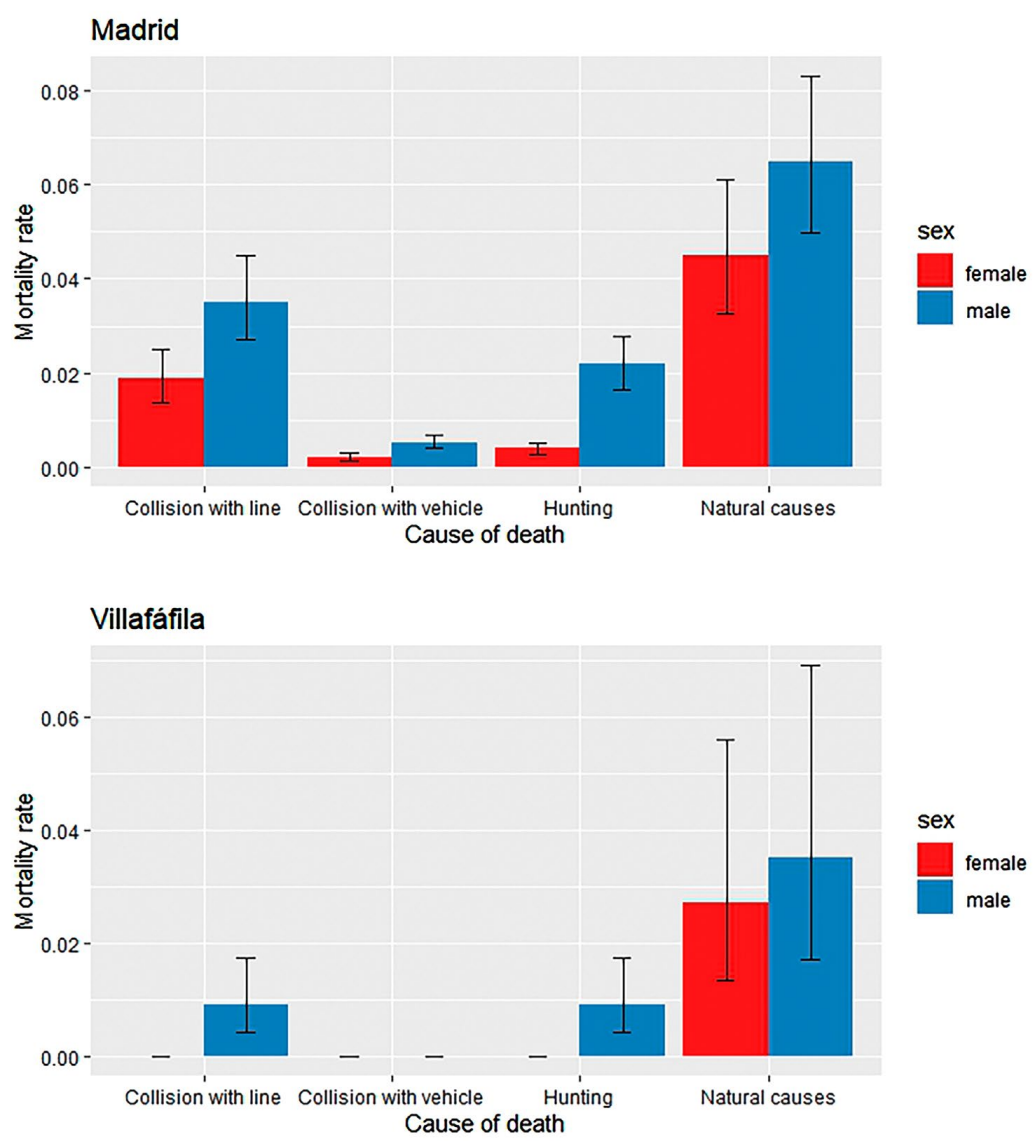
Mortality rates by sex and cause of mortality calculated from the estimates (mean and 95% CIs) derived from the most parsimonious best model S (sex+region) (model 2, Table 1) for Madrid and Villafáfila (see values in Table S5). Collision with line: power, telephone, wire fence; collision with vehicle: car, train.

A total of 118 individuals were found dead during the study (Table S5). In all study regions, the highest number of deaths was due to natural causes –which included those of unknown origin, but not attributable to any anthropogenic causes. However, anthropogenic causes reached very high proportions: 2 out of 8 (25%) individuals tagged in Villafáfila, 8 out of 26 (30.8%) in other regions and 36 out of 85 (42.3%) in Madrid (Table S5, Figure 2). The main cause of anthropogenic death was collision with power lines (26 individuals killed, 22.0% of the total number of deaths in all regions), followed by hunting (11.9%) and road kill (1.7%). In addition, one death was due to collision with a telephone line and two due to collision with wire fences (Table S5).

Mortality rates were higher in males than in females in the two main study regions (Table S6). Considering only natural causes, it was 44% higher in Madrid (0.065 in males vs 0.045 in females), and 30% higher in Villafáfila (0.035 in males vs 0.027 in females). For anthropogenic causes, the comparison could only be made in Madrid, where male mortality was 158% higher than female mortality (0.062 vs. 0.024; Table S6, Figure 2).

In Madrid, where the sample size was larger, we could confirm that the pattern of higher summer mortality recorded for both sexes in both main regions (Figure 1) was true for both only natural and only anthropogenic mortality (Figure S3). Predation, the only natural cause that we could identify in this study, had a particularly high incidence in summer (females: 3 of the 4 predation events registered in the whole year, all three at the nest; males: 2 of 4 total predation events, the other two occurred very late in the spring, just after the main display period, on 20th and 31st May; Table S7). The sex difference in anthropogenic mortality was highest in summer, when 6 males died from collision with aerial lines during their migratory movements to summer areas (Figure S3).

## 4 Discussion

The results indicate that annual survival rates were lower in males than in females across the two main study regions. In line with this finding, the maximum age reached by tagged individuals was higher in females than in males. The seasonal survival patterns were similar in both sexes, with minimum values just after the mating and incubation periods, during early summer (June–July). Although this pattern was exacerbated by anthropogenic mortality, it appeared to be primarily driven by natural mortality, which peaked in June–July. This suggests that males may be especially vulnerable immediately after mating, while females are more susceptible during incubation or just after completing it. The remarkable accumulation of deaths in both sexes during these life cycle stages leads us to speculate that reproductive processes –or the periods immediately following them– could at least partially account for these mortalities, pointing to reproductive costs as the key factor. For females in particular, predation at the nest seemed to be a relevant mortality risk. Additionally, a decline in energy reserves and immune function in late spring could make both sexes more susceptible to disease, infections and predation. As for anthropogenic mortality, it was high in both sexes, with power line casualties being the main cause of human‐induced deaths in all regions. The effects of anthropogenic mortality were more pronounced in Madrid, a region with dense human infrastructure and high anthropogenic pressure, resulting in higher annual mortality rates for both sexes than in Villafáfila, with much better‐quality habitat and less human‐induced disturbance.

Although the pattern was significant, other factors that could influence the accuracy of the estimates, such as lek or cohort, could not be evaluated in this study due to the limited sample size and the fact that most individuals were adults of unknown age. The non‐independence among individuals, however, was tested to some extent by assessing possible effects of the region and year of marking. This assessment showed that sex differences were consistent across regions and not affected by the year when the bird was captured. However, even if these fixed effects are not statistically significant, the variance component could still affect the precision of the estimates.

An important fact supporting our view that mortality stems mainly from reproductive costs is that sex‐biased mortality increases when bustards reach reproductive age compared to juvenile and immature ages. Earlier research showed that juvenile male‐biased mortality results from sex differences in vulnerability to food shortages during rapid growth, with this bias disappearing by the end of the first year (Martín et al. 2007). Its reappearance in adulthood suggests that reproductive costs could be driving the observed mortality pattern. Our findings align with previous reviews that link sex‐biased adult mortality in birds to reproductive costs (Liker and Székely 2005; Michel et al. 2022; Promislow et al. 1992; Romano et al. 2022; Sibly et al. 2012). Most studies examining the drivers of sex‐specific mortality similarly attribute higher female mortality to reproductive costs, highlighting predation during incubation as the most frequent cause (Arnold et al. 2012; Low et al. 2010; Nadal et al. 2016; Post and Götmark 2006a, 2006b; Sandercock et al. 2000; Wierucka et al. 2016; Wood et al. 2021). Other cited causes of female‐biased mortality associated with reproduction include physiological stress during breeding (Benítez‐López et al. 2015; Korschgen 1977) and death from anthropogenic causes such as mowing activities, which may affect ground‐nesting birds (Grüebler et al. 2008).

Reproductive costs are certainly high in great bustard females, as suggested by the fact that they are able to produce only one chick on average every 7–8 years, and very few manage to raise chicks in two consecutive years (Magaña 2007; Morales et al. 2002). It could be argued that, if female mortality were mainly due to reproductive costs, their period of high mortality should last several months longer than that of males, given that they raise their chicks alone and the period of dependence lasts several months. However, only around 11%–12% of females successfully complete incubation and manage to raise chicks beyond June–July (Magaña 2007, Morales et al. 2002). This would explain why the average mortality for the female sample as a whole decreases from July onwards, becoming very similar to that of males. Reproductive costs are also very high in great bustard males, as shown by several of their behavioural traits. First, their weight increases by at least 20% –probably up to > 30%– just before the mating phase, considerably more than in females (16%) (Alonso, Magaña, et al. 2009). These remarkable weight increases are among the most extreme observed in birds and have also been recorded in captivity in other large bustards (Australian Bustard *Ardeotis australis* ; Fitzherbert 1981; Kori Bustard ( *A. kori* ; S. Hallager pers. comm.)). Second, competition between males to establish a hierarchy in the lek is very strong and extends over 4 months before copulation (Alonso et al. 2010). The frequent fights, with both contenders grabbing each other by the beak and pushing each other, sometimes for more than an hour, must be exhausting and often result in serious injuries, especially on the face, as the males target the eyes of their rivals (Alonso, Magaña, et al. 2009; pers. obs.). Third, the investment is also remarkable during the mating phase, when they spend up to 10 h daily performing displays and only 14% of time feeding (Alonso, Magaña, et al. 2009; Alonso et al. 2010; Magaña 2007). Both fighting and displaying must involve a high energetic cost, which often leaves them exhausted, possibly with chronic stress and a very low immune activity, and in some cases even temporarily unable to fly, with the risk that this implies for their survival. As an example, over the study we were able to capture by hand three of these exhausted males, all in April–May, just after the display and mating period. Studies in a wide range of animals have shown that a high reproductive effort affects the immune system (Harshman and Zera 2007; Norris and Evans 2000). For example, high levels of glucocorticoid hormones associated with reproduction‐related energetic stress can suppress the immune system and increase the susceptibility to infections (Gao and Deviche 2018; McEwen et al. 1997; Ricklefs and Wikelski 2002; Sheldon and Verhulst 1996). On the other hand, the immunocompetence‐handicap hypothesis proposes that elevated testosterone levels in breeding males enhance the expression of sexual characters, but they can also directly or indirectly suppress the immune function (Owen‐Ashley et al. 2004; Roberts et al. 2004). However, this theory has not held up well under empirical testing, especially in wild bird populations (Roberts et al. 2004). In the great bustard, Bravo et al. (2014) showed that males consume proportionally more blister beetles (Meloidae) than females during the breeding season. These beetles contain cantharidin, a highly toxic compound that is lethal in moderate doses but has antihelminthic and antibacterial properties. Bravo et al. (2014) suggested that cantharidin consumption might help males reduce their parasite load, serving as an effective mechanism to compensate for their reduced immune resistance caused by their strenuous investment in sexual display, thereby reducing their post‐mating mortality.

Despite all this evidence, we acknowledge that based on current data we cannot conclude that great bustard males invest more in reproduction than females, and that this may be the cause for male‐biased mortality in this species. This would require future studies that quantitatively link individual variation in reproductive effort (e.g., mass fluctuation, display time, lek tenure in males; nesting frequency, clutch size, incubation and chick rearing success in females) to survival outcomes. Nonetheless, the male‐biased adult mortality found in our study is consistent with the reproductive cost hypothesis, particularly as outlined by Liker and Székely's (2005). Our results are also consistent with the egg productivity hypothesis (Romano et al. 2022), which shows that annual egg productivity is the strongest predictor of female‐biased mortality in birds, with mating system playing a lesser role. The investment of great bustard females in egg production is, indeed, comparatively modest: each egg weighs about 150 g, and females lay only 1–3 eggs per year (Magaña 2007), representing just 3.2%–9.6% of their spring body mass (4680 g; Alonso, Magaña, et al. 2009) –somewhat more if we account for occasional replacement clutches–. This relatively low energetic investment in gamete production may enhance female survival prospects, thereby accentuating their survival advantage over males (Romano et al. 2022), whose intense competition for mates imposes major physiological and behavioural costs. However, in contrast to most other birds, in which annual egg productivity is the strongest predictor of sex‐biased mortality and mating system has weaker explanatory power, in the great bustard the importance of these two evolutionary processes could probably be reversed, with intense male–male competition being more influential than low egg production in shaping sex‐biased mortality. In this and other life history patterns, great bustards show similarities to many mammals with high male‐biased sexual size dimorphism (Clutton‐Brock 1991; Promislow 1992). As in these, great bustards exhibit a marked male‐biased sexual size dimorphism –the greatest documented among birds and one of the greatest in Vertebrates (Fairbairn 2013)– that has likely evolved by sexual selection (Alonso, Magaña, et al. 2009; Bravo et al. 2024) and our results suggest that this could imply costs in terms of survival.

In addition to direct reproductive costs, there could also be important indirect costs. The large size males have developed to be competitive makes them more sensitive to summer heat, forcing them to migrate to cooler areas once the mating season is over, stay in the shade for longer periods than females in summer, and reduce their feeding rate, which results in minimum weights in this season (Alonso, Magaña, et al. 2009; Alonso, Palacín, et al. 2009; Alonso, Salgado, and Palacín 2016). During these summer migratory flights, males suffer additional energy expenditure and experience considerable mortality from collisions with power lines (6 of 7 anthropogenic male deaths in summer were due to power line collisions; details in Palacín et al. 2017). These early summer casualties may raise the question whether, on the one hand, the seasonal pattern in great bustard mortality and, on the other, its sex bias towards males respond more to migration costs than to breeding costs. However, several facts contradict this hypothesis. Firstly, seasonal survival patterns are identical in males and females, with minimum survival in summer in both sexes (see Figure 1), while their migration phenology is different. Males migrate mainly in summer and females in winter: in Madrid, 86% of males are migrants, the proportion varying in other regions according to latitude, with a higher proportion of migrants in southern latitudes and a lower proportion in northern latitudes; as for females, 56% migrate in winter in Madrid and 20% in Villafáfila (Alonso et al. 2000; Alonso, Palacín, et al. 2009; Palacín et al. 2009). If male‐biased summer mortality were mainly due to migration, one would expect females to also show an increase in mortality coinciding with their winter migration, which was not the case. In addition, 54% of the migrating males moved from their summer areas to wintering areas in October (Palacín et al. 2009), with no increase in mortality associated with these movements. Neither is an increase in mortality observed coinciding with the return migration to the breeding sites in either sex (September–March in males, January–April in females; Palacín et al. 2009). Secondly, the summer mortality rate due to natural causes was even higher than the anthropogenic mortality rate in both sexes, exceeding power line mortality in males (Figure S3). As for whether the sex bias in mortality would respond more to migration costs than to breeding costs, even suppressing anthropogenic mortality, the distribution of deaths of natural origin – which account for 60% of cases; see Table S5– already shows a sex bias in summer, with higher mortality rates in males (Figure S3). Anthropogenic male deaths in summer therefore contribute to enhance the male‐biased mortality caused by natural causes. In sum, both the seasonal pattern of mortality and its sex bias towards males are consistent with reproduction‐associated rather than migration‐associated vulnerability, but as we say above, cannot be definitively attributed to reproductive costs without additional evidence that relates individual reproductive efforts to survival outcomes.

The male‐biased mortality in adult great bustards is consistent with the female‐biased sex ratios observed in all populations of this species (between 1.4 and 4.1 females per male; Alonso and Alonso 1990; Alonso, Palacín, et al. 2016; Alonso, Martín, et al. 2005; www.grosstrappe.at), which are among the most skewed recorded in birds (Székely, Liker, et al. 2014). This pattern is contrary to that of most birds, in which the sex ratio is usually male‐biased, and is more similar to that of most mammals, where it is female‐biased (Donald 2007). On the basis of a review of 114 bird species, Donald (2007) concluded that higher female mortality was more determinant in the male‐skewed adult sex ratio than the skewed offspring sex ratio. Subsequently, Székely, Liker, et al. (2014), using phylogenetic analyses of 187 avian species, proposed that the most influential factor in the adult sex ratio is sex‐biased adult mortality, rather than the sex ratio at hatching or fledging. However, a more recent study based on detailed survival, fecundity, and behavioural data from a large sample of *Charadrius* plovers proposes that adult sex ratios are indeed largely dependent on sex differences in juvenile survival (Eberhart‐Phillips et al. 2018). These contrasting results suggest the need for further studies on the demographic processes that drive variation in adult sex ratios. In the great bustard, the sex ratio at hatching is 1:1 (H. Litzbarski, pers. comm., based on a sample of 531 eggs, Germany, 1979–1998), but when chicks are 2 months old it is already female‐biased, due to the higher juvenile male mortality since hatching, which occurs mainly due to starvation under food shortage (1.24 females per male, Martín et al. 2007). In adults, this sex bias seems to increase due to higher male mortality, which also appears to be the reason why females in our sample reached a maximum lifespan 3.5 years (25%) greater than males. The strongly female‐biased sex ratios in great bustards may have negative consequences for the demographic trends of most populations. For instance, an increasingly female‐biased adult sex ratio (ASR) may intensify male–male competition for mates, potentially exacerbating reproductive costs and mortality. Conversely, female‐biased ASRs could alter lek dynamics or delay female mating, with possible implications for population viability.

The sex bias in great bustard mortality is due not only to mortality from natural causes, but also to human‐induced mortality, which accounted for 39.5% of all deaths recorded in our study, and up to 43.5% in the Madrid region. Anthropogenic causes include collisions with power lines and hunting. Collisions with power lines are one of the most important causes of human‐induced mortality in birds (Loss et al. 2014), and steppe birds are particularly vulnerable to this mortality source (Bernardino et al. 2018; Marcelino et al. 2018; Martin 2011; Silva et al. 2023). In great bustards, our results indicate that collisions with overhead lines account for a mortality rate of 3.5% in males and 1.9% in females each year in Madrid. This male‐biased collision rate has been attributed to the reduced manoeuvrability in flight of males compared to females, due to their massive size and weight (Martin and Shaw 2010; Martin 2011; Bernardino et al. 2018). Therefore, this sex bias in anthropogenic mortality could be considered another consequence of strong sexual selection in this species, if we assume that the latter has led to its large sexual size dimorphism. In the case of males, collisions were much more frequent in summer (6 out of 7 anthropogenic deaths), during migration to summering areas. In a study conducted at 29 leks throughout Spain, Palacín et al. (2017) identified collisions with power lines as the main mortality cause in male migrants (37.6% of 77 birds found dead, on a sample of 180 marked adult and immature males). It was also the main cause of mortality in second‐year bustards (54.5%; Martín et al. 2007). This mortality rate due to collisions with power lines, although perhaps somewhat lower in less urbanised regions than Madrid, is probably unsustainable and is likely one of the major causes of the population declines observed in this and many other regions of Spain (Alonso et al. 2003; Alonso, Martín, et al. 2005), as well as in other countries in the species' range (www.grosstrappe.at; www.grosstrappe.de). The recent development of renewable energies in Spain is leading to the installation of hundreds of kilometres of new power lines in great bustard areas, causing collision mortality of up to 2.46 individuals per km per year (Palacín et al. 2023). These values may seriously affect the viability of some populations. Burying dangerous lines is a priority to reduce bird mortality and has been implemented in countries with highly threatened bustard populations (Raab et al. 2012). Given the importance of power line mortality in this species, future studies could model mortality as a function of proximity to power lines.

As for hunting, although it has been banned in Spain since 1980, it continued to be practised illegally in many regions and affected the first years of our study. In the two decades prior to the ban, hunting may have been the cause of the extirpation of half of the leks that disappeared in Spain (Alonso and Alonso 1996). Nowadays, it cannot be ruled out that some males may be shot, although very sporadically. Hunting mainly affected males, which were hunted and exhibited as game trophies (head and neck mounted on a plate, hung on the wall). Roadkill represents the third cause of anthropogenic mortality, with less than 1% of deaths per year in Madrid.

Although our results of differential mortality between both sexes are limited to only two regions, a relationship between mortality and adult sex ratio can be inferred. The higher anthropogenic male mortality in Madrid could likely be the cause of the higher sex bias in this region compared to Villafáfila (2.42 females per male in Madrid; Alonso et al. 2003; 1.81 in Villafáfila; Alonso and Alonso 1990). On the other hand, in Morocco, where the sex ratio was extremely biased in the past when intensive trophy hunting was practised, the male population has recovered in recent years, following the hunting ban and the decrease in poaching: from 3.85 to 4.13 females per male in 1999–2005 to 2.0–2.25 in 2019–2023 (Alonso, Martín, et al. 2005; Alonso, Palacín, et al. 2016; own unpubl. data). A similar recovery in the proportion of males following stricter hunting regulations was observed in a population of black grouse *Lyrurus tetrix* , a species in which the sex ratio is also female‐biased (Zbinden et al. 2018).

In conclusion, strong sexual selection acting on males could be the cause of male‐biased mortality in the great bustard, and the observed seasonal pattern of mortality suggests that reproductive costs could be the main cause of mortality of both sexes in this species. These patterns are enhanced by anthropogenic mortality, which is currently very high and seems to be the cause of the declines observed in most populations.

## Author Contributions

**Juan C. Alonso:** conceptualization (lead), data curation (lead), formal analysis (supporting), funding acquisition (lead), investigation (lead), methodology (equal), project administration (lead), supervision (lead), writing – original draft (lead), writing – review and editing (lead). **Beatriz Martín:** conceptualization (supporting), data curation (equal), formal analysis (lead), investigation (supporting), methodology (equal), writing – review and editing (equal). **Carlos Palacín:** conceptualization (supporting), data curation (equal), investigation (equal), methodology (equal), writing – review and editing (equal). **Carlos A. Martín:** conceptualization (supporting), data curation (equal), investigation (equal), methodology (equal), writing – review and editing (equal). **Javier A. Alonso:** conceptualization (equal), data curation (equal), funding acquisition (supporting), investigation (equal), methodology (equal), supervision (supporting), writing – review and editing (equal).

## Funding

This work was supported by Dirección General de Investigación of the Spanish Ministry of Science (projects PB91‐0081, PB94‐0068, PB97‐1252, BOS2002‐01543, CGL2005‐04893/BOS, CGL2008‐02567 and CGL2012‐36345), Instituto Nacional para la Conservación de la Naturaleza and Dirección General de Conservación de la Biodiversidad, with contributions from HENARSA, FEPMA, the Dirección General de Medio Natural of Madrid Community, and the Junta de Castilla y León, and the Consejería de Medio Ambiente, Junta de Andalucía.

## Ethics Statement

The methods used comply with the Spanish guidelines for ethical use in animal research (Spanish RD 53/2013). The study adheres to the standard guidelines for the treatment of animals in wildlife research (European directive 2010/63/UE).

## Conflicts of Interest

The authors declare no conflicts of interest.

## Supporting information


**Data S1:** ece372713‐sup‐0001‐DataS1.7z.

## Data Availability

The data that supports the findings of this study are available in the Supporting Information of this article (Table S8).
